# Reflective interventions: Enactivism and phenomenology on ways of bringing the body into intellectual engagement

**DOI:** 10.1007/s11097-020-09673-3

**Published:** 2020-05-03

**Authors:** Iris Laner

**Affiliations:** 1 Fine Arts, Art and Craft Education, University of Music and Dramatic Arts Mozarteum Salzburg, Alpenstrasse 75, 5020 Salzburg, Austria; 2 Art Theory and Cultural Studies, Academy of Fine Arts Vienna, Augasse 2-6, 1090 Wien, Austria

**Keywords:** Enactivism, Phenomenology, Merleau-Ponty, Pragmatism, Learning, Embodiment

## Abstract

When it comes to the body, the professional pedagogical field shows a paradoxical attitude: With regard to sense-oriented school subjects, educational policies tend to underline a close relatedness of body and mind. However, where learning is primarily connected with mental activities and intellectual engagement, the body is rarely assigned an integral role. Discussing the grounding ideologies of this paradox, I will consult phenomenological and enactivist perspectives in order to develop an approach to embodied learning which takes into account both sense-oriented as well as intellectual domains. I will argue that considering the interdependence of body and mind including their situatedness does not only benefit the cultivation of the body and the senses, but also opens up further perspectives for intellectual engagement.

“The word pupil has almost come to mean one who is engaged not in having a fruitful experience but in absorbing knowledge directly. Something which is called mind or consciousness is severed from the physical organs of activity” (Dewey 2008: 125).

About 100 years ago John Dewey, a pragmatist called for a rethinking of a dominant concept of learning that reduces the mind to a bodiless shell which records knowledge. As one of the most influential educational theorists of the twentieth century, he is well-known in the professional pedagogical field. He has inspired a number of reformation movements – inter alia – progressive education. Especially by focusing on experience, social impact, and individualized learning, Dewey gave impulses for the rethinking of traditional learning structures and settings (see Williams 2017, Theobald 2009).^1^ Besides, he was also one of the first distinct voices to consider body and mind as interdependent players in learning processes (see also Dewey 1958). With the elaboration of the concept of embodied cognition^2^ Dewey’s thoughts on the necessity to reframe an inherited dualistic picture of a mind that is separated from a body and, therefore able to learn independently are given a broader scientific basis. Considering cognition as embodied means that mental activities have a bodily foundation and that cognizing, perceiving, sensing, feeling, and moving are interdependent in their execution (see Varela et al. 1991, Gallagher 2005). Accordingly, learning, no matter whether it concerns bodily or mental activities, has to involve both body and mind (see i.e. Dreyfus 1987, Gallagher 2018, Brinkmann 2012, Francesconi & Tarozzi 2012).^3^

When taking a look at the concepts of learning in current professional contexts, however, it becomes debatable to what extent this insight impacts the understanding of education in the practical field. The analysis of educational policies is revealing in this respect.^4^ It suggests that being aware of the body differs greatly with regard to different domains. Generally, educational policies suggest that the more a domain of learning is dedicated to the intellect, the less important is the role attributed to the body. In contrast to this, where learning primarily focuses on the body and the senses, the mind is activated. The school subject philosophy, for instance, − the intellectual subject par excellence – is not meant to consider bodily involvement at all. Art education, to the contrary, − traditionally focusing on an education of the senses and practical skills – is regarded as one of the few canonical subjects in school education that underlines the interdependence of body and mind. This opposition hints at a paradoxical^5^ attitude: With regard to sense-oriented subjects, a close relatedness of body and mind in learning processes is underlined. Where learning is primarily connected with mental activities and intellectual engagement the body, however, is not assigned an integral role.^6^

In the following I want to address this paradox and discuss it against the backdrop of phenomenology and a phenomenologically informed branch of enactivism.^7^ Both approaches suggest that the body is indispensable when it comes to learning, no matter which kind of engagement^8^ is concerned. Generally, regarding cognition as embodied implies that body and mind are closely tied to each other and cannot be separated – neither during nor after a learning process –, since every intellectual engagement has a sensual-bodily dimension and every sensual-bodily occupation is intellectually framed (see Gallagher 2005). An enactivist understanding of embodied cognition in the sense I am referring to in this paper stresses that processes of learning involve both body and mind and are situated, i.e. have to take into account the learning environment (see Gallagher & Lindgren 2015, Gallagher 2018). Phenomenological theories, especially those which explicitly focus on the body^9^ such as Maurice Merleau-Ponty’s (2005), stress the involvement of the body in all receptive and productive acts, including intellectual ones (see Meyer-Drawe 2008, Waldenfels 2000). Moreover, educational scientists inspired by phenomenology stress the situatedness of the embodied mind not only with regard to the dynamic interaction between the learning subject and its (objective) environment, but they also underline the entanglement of the learning body with other bodies in a shared world (see Meyer-Drawe 1984, Orlikowski 2017). Considering intersubjectivity in the discussion about embodiment has consequences not only for understanding the process of learning, but also for rethinking teaching, as I will indicate when concluding the paper. Consulting both phenomenology and enactivism it can be stated that skill acquisition on a bodily level goes hand in hand with mental activities. Also intellectual engagement is considered having a bodily dimension, not only regarding the question where it stems from, but also regarding its actual practice.

On this basis it can be argued that current educational policy actually separates body and mind, hierarchizing their relationship. Herein, the different emphasis on the body in the less vs. the more intellectual subjects indicates not only the partial remainder of a dubious inherited Western dualism – an “ideology” in the sense of W.J.T. Mitchell (1986) or a “paradigm” in the sense of Thomas Kuhn (1962). In accordance with educational theorists like Dewey, it can also be suggested that such a separation inhibits learning possibilities, especially when a subject is restricted to a merely intellectual engagement that neglects the body. Neglecting the body implies not only to prevent the acquisition of bodily skills; it also curtails intellectual engagement, since the mind speaks to the body and vice versa. Consequently, a perspective towards learning that draws on the insights of phenomenology and enactivism has to discuss those educational practices critically which do not assign an essential role to the body. It has to deconstruct their inherent paradoxes and their grounding ideologies. Moreover, it should aim to point out alternative ways of dealing with learning on a theoretical and on a practical level.

My thesis considers the interdependence of body and mind, which does not only valorize bodily and sensual forms of learning, but also opens up further perspectives for intellectual engagement^10^ – in four steps. In order to give an impression of the paradoxical handling of the body-mind relation in the educational field, I will, first, take an exemplary look at the different roles ascribed to the body for achieving learning objectives by current educational policy. After that I will discuss theories of embodied cognition, phenomenological and enactivist approaches in order to develop a nuanced understanding of the role that the body plays in learning processes in general and regarding intellectual engagement in specific. I will draw on Hubert Dreyfus’ skill-acquisition model (1987), arguing that it valorizes bodily learning with respect to habitualization, but that it disqualifies the body with regard to reflection and critical engagement. Since reflection and criticality are important aspects of intellectual engagement, I will go on to discuss two accounts that offer a more fruitful perspective in this respect. I will draw on Shaun Gallagher’s insights on enactivist learning on the one hand and Maurice Merleau-Ponty’s insistence on bodily involvement in intellectual engagement on the other hand. Building upon these approaches, I will argue that a learning setting that speaks to both body and mind benefits not only skillful coping, but also intellectual engagement. It does so by avoiding an abstract, detached view and a merely formal way of dealing with a matter. Herein, it undermines a reductionist picture of reflection, criticality and intelligence. Finally, in an appendix, I will sketch how these insights allow for bringing the body into the philosophy classroom.

## The paradoxical role of the body in learning - art education vs. philosophy

In this first part, I will take a look at the Austrian national curriculum for grammar schools in order to exemplarily discuss the role attributed to the body by educational policy. I will do so by comparing sense- and praxis-oriented subjects and intellectual and theory-oriented subjects, referring to the art education curriculum on the one hand and to the philosophy curriculum on the other hand.

Art education (Bildnerische Erziehung) is a subject that builds upon a strong bodily involvement of students, both with regard to receptive as well as to productive ways of examination. The learning process initiated in the course of art education classes is defined as a “Lernen mit allen Sinnen” (“learning with all senses”).^11^ Art educators are asked to design learning situations that facilitate aesthetic experience – regarded as a synesthetic experience, affecting both body and mind. Such fields of experience are viewed as contemplative as well as creative spaces, opening up a sphere of action especially by addressing and involving the student’s body directly. The human body is referred to as a communicative, expressive means explicitly, as a medium that has to be supported in developing its motor and sensory potential. The “sinnliche Erlebnisfähigkeit” (“sensual receptivity”)^12^ is listed as one of the main educational ambitions of art education classes. Art education is not understood as being restricted to the realm of the sensual. It is supposed to affect the intellectual capacities as well, aiming at a “Vernetzung sinnlicher und kognitiver Erkenntnisse” (“interconnection of sensual and cognitive knowledge”), a merging of “Wahrnehmungsbezogenes mit Begrifflich-Logischem” (“the perceptual-oriented with the conceptual-logical”).^13^

When it comes to the role of the body, the position attributed to the school subject philosophy^14^ is quite different.^15^ Neither the body nor the senses are treated as having any effect on the learning objectives, the didactics or the general educational framework of philosophy classes. Although the curriculum lists “Erfahrungsorientierung” (“experience-orientation”) as a didactic principle, the field of experience described is restricted to the extension of the students’ lifeworld by setting up a dialogue with experts and making available extra-school learning environments. To some extent this involves bodily activity. However, the curriculum – regarded as a guide for teachers – does not urge the importance to consciously deal with the students’ bodily situation of being confronted with a new social or spatial experience and encourage them to engage with it.

Namely, the general educational objectives listed in the curriculum focus on the ability to understand, differentiate, analyze, interpret and compare. Students are also expected to learn raising and formulating questions and recognizing strengths and weaknesses, their own and those of others. The curriculum focuses on such abilities as solely intellectual abilities and herein neglects their interplay with bodily skills and sensual engagement. Such a neglect becomes clear especially where reality is addressed as one of the main learning contents to be treated in philosophy classes. Reality here is characterized as something that has to be analyzed and reflected by means of an intellectual operation that is not related to a sensual or bodily dimension.

The comparison of art education and the philosophy curriculum not only shows the different approaches to learning with respect to distinct subjects; thus by taking a look at the way they (do not) deal with the body, it also reveals two different ideas about learning. Such ideas are linked to scientific paradigms or, more generally speaking, social ideologies. As normative frameworks that imply directives for theory and for praxis such ideologies have to be contextualized and reflected regarding their enabling and restricting implications. The affirmation of the body in the art education curriculum refers to an understanding of learning as a holistic and encompassing experience. Contrary to this, the neglect of the body in the philosophy curriculum can be explained within the framework of a traditional Western dualism that separates body and mind.^16^ Since the mind is put before the body,^17^ it does not depend on the latter for acquiring knowledge, learning or engaging with a matter.^18^ Especially when it comes to intellectual tasks and abilities the body is regarded as redundant, if not obstructing.^19^ Provocatively phrased, the philosophy curriculum reflects an idea about learning that Dewey criticized in the beginning of the twentieth century as being problematic, in a practical and in a theoretical context.^20^

## The body as the Foundation of Intellectual Acts

In the history of ideas, the body enters an encompassing philosophical appreciation only in the twentieth century. Phenomenology can be named as one of the central schools of thought that underline the necessity to consider the body extensively, that not only takes into account perceptual and sensual matters. Thus, a number of accounts interested in embodied cognition are inspired by phenomenological insights, but also show interest in pragmatist concerns, like Dewey’s. On a general level, viewing cognition as embodied means that the body matters for all kinds of cognitive involvement. Namely, Shaun Gallagher’s work is located at the intersection of phenomenology and cognitive science. He underlines the bodily foundation of intellectual acts. Gallagher’s *How the Body Shapes the Mind* (2005) encapsulates the thesis that the body is not only a side-player in cognitive activities, but its very center. Gallagher considers embodiment on an explicit, i.e. phenomenal and conscious, and on an implicit, “prenoetic,” i.e. structuring and unconscious level. He states that from the moment of our birth bodily engagement in the form of “movement prefigures the lines of intentionality” (Gallagher 2005: 1). As a consequence, “*bodily* movement is closely tied in various ways to perception and to other forms of cognition and emotion” (ibid.: 8).

Considering the foundational function of the body for matters of learning, Hubert Dreyfus introduces the “Five-Stage Skill-Acquisition Model” (Dreyfus 1987). He shares Gallagher’s convictions regarding the importance of the body in perceptive, sensual, but also intellectual concerns. Stressing the mutual dependence of body and mind, his skill-acquisition model gives a nuanced understanding of the role the body plays in learning. The expert – the most skilled person in Dreyfus’ model – has incorporated rules, turned them into maxims, learned to make plans and come to decide upon the best course of action intuitively. Experts have run through a long-term learning process, ending up in being able to spontaneously respond to situations without having to reflect and analyze them. They are capable of acting out their expertise immediately. According to Dreyfus, an expert practice is not a reflective practice. Rather, it is a habitualized way of doing something that has proven to be the right way over time.^21^ The body is understood as the core player in the learning process, since learning in the sense of habitualization is regarded as dependent on recurring, lived experience.

Dreyfus stresses that experts often cannot give an account of the best course of action, i.e. they cannot verbalize their expertise. For him, this indicates that skillful coping in experts is non-conceptual.^22^ Experts have learned to adequately respond to a situation intuitively and immediately, i.e. without the need of stepping back and thinking^23^ before acting. Also regarding intellectual expertise in the field of moral judgment and action, Dreyfus claims that learning to cope with problematic situations cannot be achieved by detaching oneself and studying principles of the right course of action on an abstract level (Dreyfus & Dreyfus 2004). The mind alone cannot provide the basis for judgment and action here. Rather, as in regular practices, intellectually challenging situations have to be experienced and lived through. Rules have to be discovered and incorporated. Also, they have to be tested in various situations where they can be contextualized and relativized. Maxims start to give orientation for judgment and action. In the course of exploring diverse contexts, situated analysis and reflection one gradually becomes an expert, finally being able to judge and act intuitively, immediately, without any need of stepping back, thinking through and further learning.^24^

In any case, Dreyfus takes the expert’s position to be a skilled view that stems from a bodily engagement in the world. With regard to intellectual expertise, however, there is a catch^25^: The unreflective way of expert acting might come along with a tunnel vision, i.e. a specialized expertise that is limited and cannot reflected regarding its limitations, since the expert has reached a stage beyond further learning. When it comes to dealing with complex and ambiguous situations, the expert’s unreflective and intuitive way of doing things becomes problematic. In such cases expert practices have to be revised, opened for reflection and, eventually, relativization. For Dreyfus, this means that “the wise, intuitive decision maker will attempt to dislodge his or her current understanding” (ibid.: 256). Practically, “dislodging” one’s expert understanding can be achieved by re-experiencing certain stages on the way of becoming an expert, entering a dialogue with other experts and trying to take over other perspectives (ibid.: 256 f.). At the level of intellectual engagement, it is thus necessary to confront habitualization with strategies of “unlearning,”^26^ as I suggest to phrase it. This is because skillful action, which is characterized as incorporated, practical, unreflective, intuitive and immediate, fails to meet the intellectual challenge of dealing with complex and ambiguous situations. In the light of intellectual engagement the Dreyfusian expert therefore has to re-enter the process of learning, coming back to a lower stage of skill-acquisition, revising the acquired bodily knowledge which initially gave them the ability to judge and act intuitively.

Although Dreyfus therefore valorizes the role of the body in processes of learning by defining expert knowledge as ultimately practical bodily knowledge, his argument seems to involve a dualistic conception of body and mind^27^ regarding questions of learning. The body is introduced as the normalizing, unreflective, intuitive player in the learning process. It incorporates standards and rules, developing the ability to respond to new situations intuitively, but still in an adequate^28^ way. The very ability to respond intuitively in an adequate way is pushed to its limits where complexity and ambiguity comes in. Here, the Dreyfusian body fails to respond to new challenges and has to face its counterpart – the mind – as the reflective, non-intuitive player.^29^ In this dualistic set up the mind lacks the ability to intuitive response, but it is the one to remind the body to face its own limitations, taking over the task of reflecting upon the complexity of the situation.

The Dreyfusian approach, which regards body-oriented forms of learning as habitualization that is in need of further correction as soon as unusual, problematic or ambiguous situations are discussed, underlines the bodily foundation of all ways of skillful coping, including intellectual engagement. Herein, it helps to understand how embodied, incorporated kinds of knowledge and expertise work as a corrective to abstract and detached forms of intellectual engagement. However, when Dreyfus suggests that skillful coping becomes problematic in the light of intellectual challenges, he re-introduces the body-mind dualism^30^ which takes the body to be the unreflective, sluggish, and passive instance. Here, the subject has stopped to learn and basically refers to solutions already known; the mind is viewed as the reflective, flexible, and active player reminding of the necessity to still learn^31^ and to create new solutions^32^ in the engagement with the world. With regard to intellectual challenges this dualistic set up is problematic, because it reserves the role of reflection and critical involvement^33^ to the mind. If in the discussion of learning processes the importance of the body has been limited to skillful coping in terms of unreflective action, it is difficult to affirm the significance of explicitly appreciating the body not only in the more sensually oriented, but also in the more intellectually oriented kinds of engagement. Moreover, if the importance of the body is stressed with regard to its foundational qualities^34^ only, reflection and critical involvement become a unique feature of the mind. The body, however, not only provides the basis for reflection and acting critically in complex and ambiguous situations; it also shows active participation in reflective and critical practices^35^ and helps to introduce a situated understanding of these terms. Criticizing Dreyfus’ dualistic set up of the unreflective body and the reflective mind regarding intellectual challenges, I argue in support of a situated reflection and critical involvement which allows for understanding the intellectual engagement of an embodied mind that is not detached, but embedded.

This is because, responding to the paradox of the different role ascribed to the body in different domains of learning, as referred to above, one needs to consider not only the foundational role of the body, i.e. skill-acquisition in the form of habitualization.^36^ In order to spell out my argument concerning situated reflection, I finally want to turn to Gallagher’s enactivism and Merleau-Ponty’s phenomenology as underlining the importance of habitualization as well, but as giving yet another idea of considering the more active role of the body in intellectually challenging situations. Here, the body can be regarded as the instance which reminds the mind that it still has to learn, i.e. be open for change in the light of intelligently^37^ responding to new situations. Both authors focus on the body as an implicit as well as an explicit player in the learning process. Consequently, the body appears not only as the foundation of intellectual acts, but as a critical corrective to a reductionist understanding of acting reflectively and critically. Bringing the body into intellectually compelling situations as an active and flexible player helps to multiply and understand diverse perspectives on the same problem, reduce detachment and abstraction, relativize intellectual reflection that is not in line with the life world and refer to the practical implications (on a perceptual, sensual, emotional etc.) level of thoughts and judgments. With taking over the perspective of enactivism and phenomenology, I want to frame a possible scenario for stressing the importance of the body for intellectual engagement, not only as constituting its foundation, but also as allowing for a situated reflection and critical involvement here and now.^38^

## Enactivism and phenomenology on possibilities of bringing the body into intellectual engagement

When it comes to matters of learning, Gallagher holds an enactivist approach.^39^ Besides underlining the entanglement of body and mind, enactivism stresses their situatedness, i.e. the relatedness to their respective environment (see Varela et al. 1991).^40^ Body, mind and environment are mutually dependent, since experience is action-oriented. Action-orientation implies that the structuring, self-defining phrase of a subject is “I can.”^41^ This means that the constitution of the subject is linked to specific situations that open up possibilities for action. Accordingly, to make an experience and to learn are regarded as dynamic processes in which an embodied mind interacts with its environment.

Like a range of accounts with interest in embodied cognition in general, Gallagher’s enactivist approach draws on phenomenological insights. With its emphasis on action it also shows a pragmatist orientation (see Gallagher 2014). It is therefore not surprising that Gallagher’s concept of learning overlaps with Dewey’s in some core respects. Gallagher underlines that a learning situation encompasses the embodied mind and its situation. Since they are interwoven in a dynamic way, altering one element necessarily affects the others. For (re-)structuring a learning setting this means that one has to consider each of the elements involved per se *and* in relation to the others. Accordingly, “intervention on bodily or environmental factors will also indirectly be an intervention on the brain. Plastic changes in the brain come along with changes in bodily habits or changes in experienced environments” (Gallagher 2018: 635).

Similar to Dewey, Gallagher criticizes educational settings that regulate the body and neutralize the situation of learning. According to him, it is not unusual that educators fall back into “think[ing] that bodily movements and environmental arrangements are not relevant to intellectual development, or are, at best, enabling conditions but not the right stuff that we need to educate” (ibid.: 635). Therefore, he brings in empirical evidence on pretend plays and “learning scientific principles through whole-body immersion in mixed reality simulations” (ibid.: 641) that affirm the positive effect of learning settings that recognize the mutual dynamic connections of body, mind and environment (see also Gallagher & Lindgren 2015). With this emphasis he goes beyond Dreyfus’ claim that the body has to be appreciated as the foundation of intellectual acts. In my reading, referring to pretend plays, Gallagher suggests that the body is attributed a more active and reflecting role in intellectual engagement. In order to elucidate on what I mean by a more active and reflecting role of the body, I will finally turn to phenomenology to complete my argument regarding situated reflection.

Gallagher’s enactivism is strongly influenced by phenomenology. Maurice Merleau-Ponty is one of the main sources of reference.^42^ This comes as no surprise since Merleau-Ponty not only shares the common interest in the dynamic entanglements of body, mind and environment. Above that, like many working in the field of embodied cognition, he engages with science.^43^ Merleau-Ponty’s rapport with science allows for drawing inferences about the dialogue not only between theory and empirical research, but also between intellectual and bodily engagement, between intelligence and skill.^44^ Merleau-Ponty’s 1956–7 course on “The Concept of Nature” (Merleau-Ponty 2003) – especially his lectures on “Modern Science and Nature” – is illuminating in this respect, since it hints at the possibilities, if not the necessity, of philosophy to take a leaf out of the book of modern physics. It is interesting for my concerns here, because this leaf describes the very corporeality and situatedness of the scientist in the process of scientific investigation. Herein, it extrapolates the dynamic correlation of body, mind, and environment in a problem-centered learning setting. This setting exposes a kind of intellectual engagement that is inseparable from bodily and sensual activity.

Merleau-Ponty is convinced that modern science involves the body consciously and reflects upon the down-to-earthness of its engagement not in a detached, but in a situated way. Accordingly, what philosophy can learn from science is to engage more directly with phenomena. This means that philosophy has to proceed in a less detached way, starting to intervene.^45^ Modern scientific praxis builds upon the recognition of the fact that every observation must be related to its situation. This underlines that within an experimental setting, which can be regarded as an engagement with the world where – to quote Ryle – an expert “is still learning” (Ryle 1990: 42), modern physics regards it as indispensable to reflect upon^46^ the very body which observes and, in this sense, intervenes. Although Merleau-Ponty does not deal with the question of learning explicitly, this passage is interesting in order to understand the necessity of an active involvement of the body in intellectual ways of engagement.

Merleau-Ponty states that the philosopher should follow the physicist’s example when it comes to the question of how to deal with phenomena. Although the philosopher’s business might be abstract, they still have to make sure to stay grounded. In his early work, Merleau-Ponty also refers to the necessity of grounding philosophy, but more in terms of reflecting contents. Loosely speaking, the way the philosopher deals with phenomena is a reflection upon theories and empirical findings proceeding in a dialectical way. While Merleau-Ponty’s phenomenology of perception deals with the empirical sciences dialectically (see Merleau-Ponty 2005: 326), in the 1950’s he develops a more critical approach towards scientific knowledge in general until he finally comes to reject it, largely in his last essay *Eye and Mind* (1993) and, partly in his unfinished work *The Visible and the Invisible* (1968b).^47^ The reason for this rejection can be seen in his growing interest for ontological problems. This “ontological shift” is connected with the search for an alternative philosophical method.^48^ As Being can neither be described nor conceived, it finally seems impossible to empirically investigate or to reflect upon it. The detached view has become even more problematic.

For Merleau-Ponty, one way to cope with ontological questions is to focus on what he designates as “primordial experience.”^49^ Primordial experience is a mode of experience that is more profound and thus more close to “brute” or “wild” being in which Merleau-Ponty takes interest in his later phenomenology (see Merleau-Ponty 1968a: 168ff.). Brute or wild being is the being grounding the beings in the world and is closely connected with his understanding of nature. Both refer to an unorganized, unstructured level of experience that we are unfamiliar with as we normally organize and structure our experiences. It is due to this determining striving to order things that it becomes nearly impossible to grasp the phenomenal appearance of nature.^50^ Brute being cannot be grasped by a habitualized, ordering and structuring way of experience. It can neither be grasped by a detached mind, nor can it be analyzed by ways of intellectual operation. First and foremost, it must be lived, bodily engaging in a situation. Here, however, it naturally slips the mind as well as the habitualized body.

How can the philosopher unlearn to engage with phenomena in a habitualized and a detached fashion at the same time and start to intervene? How can one stop to deal with things in a normalized fashion and arrive at an intellectual engagement without forgetting about the situation one is in and without leaving the body aside?

In order to answer this question, Merleau-Ponty suggests to take a look on how modern science criticizes itself: “Science is not an unmotivated instance. We have to psychoanalyze science, purify it. Scientific consciousness lives in the natural attitude, as Husserl said, and it ignores Nature because it is there: it is a naive and uncritical enjoyment of the natural certitude. […] But modern science often criticizes itself and its own ontology” (Merleau-Ponty 2003: 85). Modern science criticizes itself when it questions the relation between the scientist and the scientific object – the observer and the observed. According to Merleau-Ponty, it does so whenever it is “at work,” meaning when a scientist is in the course of investigating phenomena. Since “the position of the philosopher is not without risk” (ibid.), they have to “unlearn” by following the example of the scientist. Structurally, the way philosophers have to unlearn is not entirely different from Dreyfusian’s expert way of learning when they face an ambiguous situation. However, for Merleau-Ponty the instance that reminds of the need to still learn, i.e. be open for the actual challenges of the situation here and now and respond to them, is the body and not the mind. In the Merleau-Pontian picture, the expert – the philosopher – has to stop dealing with problems the way they are used to. They have to interrupt their expertise. The critical corrective that comes in to break up a habitualized action is not the detachedly reflecting mind, but it is the intervening body.

While the philosopher is eager to “see” – i.e. to understand the matter of their research – the scientist starts their work by engaging with the matter at hand. Merleau-Ponty states that the scientist seeks to intervene, i.e. to get actively involved in the process of examination: “The concern of the philosopher is to see; that of the scientist is to find a foothold. His thinking is directed by the concern not of seeing, but of intervening. He wants to escape getting bogged down in the philosophical way of looking at things. Does he also often work like a blind man by analogy? Did a solution work out for him? He tries it on something else, because that time it was successful. The scientist has the superstition of means that succeed. But in this attempt to get a firm grip on things, the scientist discloses more than he sees in fact. The philosopher must see behind the back of the physicist what the physicist himself does not see” (Ibid.: 86–7). The difference between the philosophical and the scientific attitude, as Merleau-Ponty stresses it in this passage, is that approaching a problem philosophically one does not engage with the matter of interest, but touches on it from a reflective distance, while the scientist plunges into it. Unlike the philosopher, the scientist does not start from a detached perspective, by trying to understand, but they begin by doing something, i.e. arranging a scientific setting and observing the scientific object that they take interest in. In the process of investigation they proceed by sticking to experimental principles. If something works out they move onward; if an experimental set-up does not produce the output intended they design a different one. In engaging with the matter directly, the scientist faces an actual experience, while the philosopher merely tries to grasp the phenomenon without experiencing it. However, the scientist is not acting blindly, but reflects on what they are doing. They do so, not in a detached, but in a situated sense. The body is the critical corrective that comes in to stay grounded, to take over this perspective or another, to multiply concrete views and approaches, to live, feel, and experience the relationship between researcher and the matter of research.

Merleau-Ponty’s argument concerning the situation of the philosopher is probably intended to criticize the more academic way of doing philosophy. However, there are some aspects that I would like to single out in order to think about the role of the body within intellectual engagement in a way that cares about the body and the situation to the same extent as it cares about the mind. A very general consequence that can be drawn from Merleau-Ponty’s approach is that reducing intellectual engagement to a detached thinking through abstract problems risks to lose the relation to the world one lives in and to what ultimately matters. When he states that the philosopher should not rush to see, i.e. to take over a detached perspective from the very beginning, he stresses that there is a need to take the process of orientation in a situation seriously. In order to gain orientation it is indispensable to experience the situation as a field of possibilities. For Merleau-Ponty, as for Gallagher and Dreyfus, experience is not a passive form of suffering, but it is action-oriented. Experiencing here implies to be ready to intervene. Being ready to intervene a philosopher has to engage with a situation. They have to analyze, reflect, and deconstruct as well. But they have to do so while being at work, i.e. they have to do so in a situated way. Stressing intervention and being at work underline that the body has to be brought in on a conscious level, namely as an active player in a problem-centered learning setting. The body needs to ground the mind, but not only in terms of being its general foundation, but also in terms of a critical corrective that helps to find a foothold, gain perspective, develop an engaged, and situated approach in an intellectually challenging situation. Once the body is understood not only as the foundation of intellectual acts, but as an active player in intellectual engagement, the dualistic set up of body and mind including the hierarchies it brings along is questioned. Then, it is not the mind that is brought into body- and sense-oriented learning processes in order to valorize them. Rather, also the body can be brought into intellectual engagement in order to reduce detachment and abstraction, in order to call for a critical engagement that matters in the world.

My argument concerning situated reflection and critical involvement of the body, like every thinking, is bound to an ideology as well. However, it is worth to thematize the framing, the possibilities and limitation of such an ideology, as well in order to be able to actively decide which one is in line for a society that is perhaps in need of less detachment and which is more situated and engaged strategies to cope with the actual challenges in the world.

## The body in the philosophy classroom

I will conclude by giving a concrete example of an intervention of the body in a learning setting that focuses on intellectual engagement. In order to do so, I want to come back to the case of learning in schools.^51^ How could a philosophical space for intervention be set up in the classroom? How could it be transformed into a learning setting that calls for an intellectual engagement that does not neglect, but consciously integrates the situated body?

Considering the restricted spatial and temporal opportunities of doing philosophy in school, one cannot expect the students to become experts in a Dreyfusian sense. His approach is not useful for reflecting learning settings in schools, since school learning has limited time and space resources which are dedicated to studying distinct subjects. Accordingly, Gallagher’s position not only helps to stress the importance of bringing the body into schools, it also gives a concrete example of doing so. His example focuses on science classes, though. In science classes it is quite common to create experimental learning settings. With respect to philosophy classes I regard it as more fruitful to consider the issue of pretend plays Gallagher brings in without talking about the positive impact it might have on learning in schools. Experience in the mode of “as-if” provides a huge set of opportunities to engage with situations that are problematic and intellectually challenging. Role plays, enactments of moral dilemmas, but also engaging with literature, films, theatre or music can open up experiential spaces for a situated, body-sensitive philosophical engagement.^52^

With reference to the Scottish performer Chris Thorpe I suggest to consider the philosophy classroom as a “laboratory for thinking about how to think”.^53^ Thorpe regards the theatre as such a kind of laboratory where an experiential space is created. Examining how we think about confirmation bias, the performer deals with a challenging philosophical issue by engaging bodily with a problematic situation. He thinks about how we think by ways of performing a dialogue between himself and Glenn, a white supremacist, he interviewed in preparation for the play. The audience taking part in the performance is asked to intervene as well, in the mode of “as-if.” How would you act if you were in the situation of discussing sensitive moral issues with a white supremacist? How would you behave? How would you speak? How would you argue your case? Thorpe’s laboratory involves body and mind, perception, emotion and intellect. It allows for experiencing their interconnectedness. But this is not all: It also calls for intervening in the sense of trying “to find a foothold,” (Merleau-Ponty 2003: 86) not only in a process of trial and error, but also by entering a stage of (self-)reflection. Such reflection underlines the need to take over other perspectives in order to better understand what is going on. So it does not call for a detached view, but for a situated view from different perspectives, taken over directly as well as in the mode of “as-if.”

Creating a laboratory of thinking about how we think in the classroom, i.e. an experiential, experimental, affective, emotional, moving space that exposes a problem and invites to engage with it can be achieved without much effort.^54^ Pretend plays can help to take over different perspectives. They can be initiated with the help of role plays, pictures, novels, comic books, movies, TV-shows and many more.^55^ Working with materials out of the students’ lifeworlds can encourage them to make use of an intellectual engagement that is mindful of the body also beyond the philosophy classroom.

What is needed in order to give way to create such spaces in schools, is a restructuring of learning settings. Such a restructuring comes along with a range of political, social and also pedagogical challenges.^56^ In order to deal with such challenges a reconception of what it means to engage with a matter on an intellectual level is needed. Once such a reconception is on its way to challenge existing educational praxis and policies, philosophy in schools can give students the possibility to learn to engage with a problematic, intellectually challenging situation in the course of a situated, body-sensitive analysis, namely an engaged intervention. The kind of reconception that allows for engaged forms of interventions in the philosophy classroom has to be bound to reflecting the role of the learning body in school more generally. In order to raise awareness for the complex entanglements of body and mind in learning processes in a more encompassing sense it is not enough to bring in alternative learning settings, since they affect not only students, but also teachers: Once not only the mind, but the body of the learner is recognized as learning it becomes even more important to think about the sharing of a space with the students, of being exposed to the same experiential setting. Accordingly, the classroom has to be designed as a place of an encounter where teachers and students interact and engage with the matter of interest in a cooperative way.

## Footnotes

1 Referring to the educational systems in the US, Theobald (2009) states that Dewey’s influence in the past was considerable. Nowadays, however, there is a reversion to what Dewey regarded as ‘traditional’.

2 In this paper, I am referring to a strand of discussion about cognition and embodiment that mental activities cannot be understood without considering the co-constitution of body and mind. Embodied cognition is a concept that has come up in countless debates recently, since the conviction is widely shared that mental activities cannot be fully separated from bodily and sensual activities. The argument developed in this paper does not intend to contribute to or touch on all these debates. Rather, it is directed towards those approaches that build upon the phenomenological idea that every way of being in and relating to the world involves both body and mind (see Merleau-Ponty 2005).

3 Besides Dewey’s pragmatist approach and Merleau-Ponty’s phenomenology, Gilbert Ryle (1990) and Michael Polanyi (1966) bring in other early perspectives that underline the importance of thinking about the relatedness of body and mind in learning processes.

4 School curricula set the scene for institutionalized learning. Therefore it is of interest to study educational policies in order to track a current, socially defining understanding of learning. Interestingly, the idea of an entanglement of body and mind is totally out of the scope of some school curricula, as I will show below.

5 The attitude is not that paradoxical if one considers the hierarchy implied: The mind stands above the body. In sense-oriented subjects the interdependence of body and mind is utilized to valorize the merely bodily and sensual domains of learning. In the intellectual domain, however, such a valorization is not needed; therefore, the body is not brought into the discussion.

6 Learning here refers to a process of change (regarding skills, knowledge, but also attitudes) that can be understood in at least two different ways: 1. in terms of growth, i.e. a positive accumulation of experiences (Dewey 1958), 2. in terms of irritation, i.e. the confrontation with “negative experiences” (Benner 2005) or “Widerfahrnis” (Meyer-Drawe 2005) that necessitates a reorientation. Regarding intellectual engagement learning can therefore mean that one acquires the skills, the knowledge and the attitude that are indispensable for intellectual engagement. It can also mean that one is exposed to irritations or “Widerfahrnisse” that call for a reorientation. Although the latter case might seem eventful and speak only to the very moment in which one is irritated, it can also elicit a sustainable learning process, since it reminds the learner of the necessity to still learn.

7 In philosophy and cognitive science, there is a range of different approaches considered enactivist. By speaking of enactivism I am referring to accounts that focus on the actual entanglement of the living body, the mind and the environment. I am not referring to accounts that concentrate on the more abstract idea that cognition is an active engagement with the world without explicitly thematizing the role of the living body in this context.

8 In this paper I am using the Dreyfusian term skillful coping (see Dreyfus 2014) in order to describe bodily and sensual engagement; I am using the term intellectual engagement in order to describe ways of dealing with a matter that do not integrate the body or the senses in any obvious way.

9 Even before Merleau-Ponty’s (2005) claim to consider the embodiment of mind and consciousness, Edmund Husserl stresses the role of the body (see e.g. Husserl 1952, 1985). Heinämaa et al. (2014) argue that for Husserl the body is not only a practical, but a transcendental necessity.

10 Focusing on intellectual engagement might seem problematic, since my paper aims to question a dualistic understanding, separating body and mind. Although I find it important to stress the interdependence and relatedness of body and mind on multiple levels, I regard it as essential to work with differentiations for analytical purposes. My focus on intellectual engagement has a thematic, but also a strategic reason. Many educational studies in the field of embodied cognition concentrate on more bodily and sensual forms of learning, leaving the issue of intellectual engagement aside. See i.e. Brinkmann 2012.

11 https://www.ris.bka.gv.at/GeltendeFassung.wxe?Abfrage=Bundesnormen&Gesetzesnummer=10008568 (accessed: 11.12.2019)

12 In the Anglo-American discussion, it is also described using the term “percipience” (Smith 2008).

13 Already very early approaches in aesthetics (see Baumgarten 2007) underline this kind of merging in the course of aesthetic engagement.

14 In the Austrian national curriculum for grammar schools philosophy is not a separate subject. Together with psychology it forms the composite subject psychology and philosophy (*Psychologie und Philosophie*), psychology being taught in the 7th grade and philosophy in the 8th and final grade of grammar school.

15 However, some parallels are remarkable: Art education and philosophy both play an essential role for developing a reflected and critical rapport with the students’ life-world.

16 René Descartes (1999) is often listed as one of the most influential thinkers to introduce the idea of a separatedness of body and mind.

17 As I said before, one could argue that also the valorization of a body- and sense-oriented subject like art education by ways of underlining its cognitive impact affirms a hierarchy of mind and body where the mind comes first.

18 Even more radical, according to Descartes (1999), the mind can refer to itself without even needing to appeal to a body in order to consider the essence of subjective existence (“I think, therefore I am”). In the Cartesian picture the body is thus often described as a burden of the mind when it comes to intellectual growth.

19 The discourse about critique, which disregards the body as counterproductive for critical engagement, adheres to such a picture (see Arendt, 1959, Kant 1998). Judith Butler (2015) or Gayatri Spivak (2012) are the few exceptions who introduce a notion of critique that considers the body in an affirmative sense. For further elaboration on a bodily dimension of critique see Laner 2019.

20 According to Gallagher, such an understanding is underpinned by a “standard cognitivist (representational) approach” (Gallagher 2018: 631), which conceptualizes “learning as primarily an intervention on the brain” (ibid.). Although this notion might seem outdated it is still very influential in educational thinking nowadays.

21 In this respect, Dreyfus’ argument is close to what Wittgenstein says about expert practice in his *Lectures on Aesthetics*. Rietveld (2008) underlines that Wittgenstein’s understanding of expertise amounts to an everyday practice that still involves a basic kind of normativity: situated normativity. Rietveld (2010) also stresses that Dreyfus’ account is similar to McDowell’s, since both focus on “unreflective action,” i.e. habitualized practices that allow for coping with issues in everyday life in an adequate way without reflecting about the respective adequacy.

22 Dreyfus outlines that Plato often enters a dialogue with experts who do not have a clue about what they are actually doing; “[e]xperts, at least in areas involving non-empirical knowledge such as morality and mathematics, had, in another life, learned the principles involved, Plato said, but they had forgotten them.” (Dreyfus 1987: 20) McDowell (1996) also regards skillful action as second nature, i.e. as habitualized. Still he underlines that there is a conceptual level in unreflective action. Compared to McDowell, the Dreyfusian account can be regarded as rigorous.

23 In Dreyfus’ understanding stepping back and thinking can be equated with reflecting.

24 Interestingly, for Dreyfus, once somebody has become an expert they are not dependent on further learning, since they “have gotten past the learning phase” (Dreyfus 2005: 51). Opposed to this, Ryle suggests that intelligent practices that display knowing-how, i.e. incorporated, bodily knowledge, are characterized by an openness and flexibility of the practitioner to change their approach eventually: “It is of the essence of intelligent practices that one performance is modified by its predecessors. The agent is still learning” (Ryle 1990: 42). In Ryle’s understanding Dreyfus’ expert, who is not learning anymore, is a person not acting intelligently, but merely habitually.

25 In line with Dreyfus, Rietveld (2010) underlines the importance of appreciating unreflective expertise for its own sake, since it plays a very important role in everyday life. I do not want to question the importance of unreflective action or expertise. However, I think it is crucial to think about possible transitions between something like an entirely unreflective action and reflection, since the rigorous opposition between reflective and unreflective risks to repeat the dualistic logic of mind and body.

26 I am referring to Michel Foucault’s (2001) use of the term “désapprendre” here. This term is taken up by more recent approaches (see Chokr 2009) that deal with strategies of questioning normalized views.

27 Berendzen (2010) argues that Dreyfus’ foundationalism is not only dualistic regarding questions of learning, but generally understands coping as opposed to intelligent engagement.

28 For becoming an expert it is not only key to act in the way that has proven to be right in the past; an expert also needs to be sensitive to what is challenging about a new situation and act accordingly. See Dreyfus 2005. This sensitivity seems to have limited effectivity though, since in the case of complex and ambiguous situations the incorporated expertise fails to respond to the newness of the situation. This is when the ability of the mind to reflect comes in again, opposing the body’s unreflective coping.

29 According to Dreyfus, regarding intellectual issues it is not wishful, but it is possible to engage in a purely rational, detached fashion: “None of the above is meant to deny that an ethical situation could occur so unlike any previous situation that no one would have an expert intuitive response to it. Then, no amount of involved deliberation would serve to sharpen the expert’s intuitions. In the face of such a total breakdown, and in that case alone, the ethical expert would have to turn to detached reflection” (Dreyfus & Dreyfus 2004: 261).

30 There is a range of conceptual pairs that come up in recent discussions in order to balance common and distinct features of body and mind. It would also be interesting to think about the differences I am referring to in terms of conscious/unconscious and aware/unaware, regarding the bodily exercise of an expert practice as unconscious and – at least to some extent – unaware of some circumstances and its own limitations. Although these concepts are crucial in phenomenological and enactivist discussion, I will be focusing on the pair reflective/unreflective, since in my understanding it most directly serves my purpose of elaborating on the role of the body in intellectual engagement.

31 One could even argue that Dreyfus’ five-stage-model suggests that the mind, which is associated with the ability of stepping back, reflecting and critically investigating in his dualistic set up, motivates the body to learn: As soon as there is no involvement of the mind in the learning process, the body stops to learn.

32 It would also be interesting to discuss the aspect of creativity in this respect. Creative practices might indicate a kind of skillful action that is set in between expertise as incorporated knowledge and openness to reflection, critical evaluation, and change. Since creativity is a big issue I have to leave this aspect to elaboration elsewhere.

33 In my understanding reflection cannot be reduced to intentional, reflective thought. Neither can criticality be reduced to the verbal utterance of a verdict. Reflection means more than to relate to something propositionally and by ways of thinking it through. It also means to ponder. Now, one can ponder by way of using propositions, concepts or words. However, one can also ponder by way of contemplating, sensing, in living relation to oneself and a matter. I suggest to use the term situated reflection in order to describe such instances of grounded, non-detached, involved ways of reflection.

34 The foundational qualities are key. I do not intend to question the significance of considering the foundational level of incorporation and habitualization. Still, I think it is crucial to consider also moves of bodily engagement that show reflective potential and could help to dismantle the rigorous dualism of unreflective, sluggish bodies and reflective, flexible minds.

35 Ryle (1990) underlines this, when he claims that an intelligent performance stems from incorporated knowledge, but is still flexible and open for response. In his understanding, the intellectual engagement is not something else, but is part of intelligent performances, denying “that the execution of intelligent performances entails the additional execution of intellectual operations” (Ryle 1990: 48). Herein, he actually aspires to dissolve the dualistic set up and hierarchical order of body and mind.

36 Deconstructing Dreyfus’ use of Merleau-Pontian ideas, Berendzen (2010) stresses that Merleau-Ponty’s understanding of sedimentation is more encompassing than Dreyfus suggests. It is not restricted to bodily and sensual skills. It also comprises the sedimentation of thoughts, ideas, beliefs, phantasies etc. Taken this way, sedimentation hints at a deep entanglement of body and mind and not at their separation in terms of making one the foundation of the other.

37 Using the term ‘intelligence,’ I am referring to Dewey and also Ryle who underline that intelligence has to be understood in an encompassing sense that does not exclude bodily and sensual ways of dealing with a matter. Dewey regards artistic practice as intelligent, because in the course of the performance a skilled artist develops a new expression (Dewey 1958: 90). The artist finds new ways of doing something not by reflecting, but by experimenting (ibid.: 149). Ryle (1990) describes expert practices as intelligent and critical, since, in his understanding, an expert might not be able to give an account of the way they proceed, but is still able to respond to a situation flexibly. Gilbert Ryle (1990) offers an intriguing perspective as well when it comes to the discussion of skill and intelligence. Using the term ‘intelligent’ to describe bodily performances that indicate “care, vigilance or criticism” (Ryle 1990: 42), he hints at a form of skillful action that is different to a mere habit. More than discussing the connections of knowing-how and knowing-that, Ryle is interested in the epistemic value of skills..

38 I owe my thoughts on intelligent interventions as a form of engaged critical intervention that is not detached, but conducted out of a situation that affects the performer directly also to Antonio Gramsci and Chantal Mouffe who are not the center of attention here.

39 For the diversity of enactivist approaches see Ward et al. 2017. There are enactivist approaches that do not focus on the entire body, but on sensorimotor abilities, such as Noe (2004). However, Gallagher’s enactivist approach runs in the tradition of Varela et al. (1991) in working with insights from Embodied Cognitive Science and traditional phenomenology. In this paper, I will refer to approaches interested in embodiment that share the conviction that it is necessary to thematize the entire living body in its actual surrounding.

40 At this, *The Embodied Mind* (1991) is often regarded as a standard publication for both the fields of Embodied Cognitive Science as well as Enactivism. While the theory of embodied cognition – in a very general way – focuses on the entanglement of body and mind, enactivism takes into account the situatedness of body and mind as necessary conditioning of the relatedness of body and mind.

41 In the Western history of ideas, the classical self-defining phrase is “I am” or “I think.” This phrase reduces the subject to its (mental) self. Developing a different conception of self and subjectivity as genetically formed and dynamically interacting with its environment goes back to Husserl (1950). Merleau-Ponty (2005) builds upon Husserl, using the term “I can” to describe subjectivity as a bodily being in the world. With his theory of affordance Gibson (1977) appeals to this idea. He is an important precursor of the enactivist movement as well.

42 Gallagher names Merleau-Ponty as one the core sources of inspiration. Also Dreyfus references Merleau-Ponty and other phenomenologists, like Heidegger.

43 While the early Merleau-Ponty builds upon scientific findings (Merleau-Ponty 1967 & 1968a), his later writings advance a less affirmative approach, moving towards a more general methodological shift.

44 Merleau-Ponty is one of the phenomenological authors who is often referred to in educational theory. Normally either his theory of embodiment and sedimentation or his thoughts on intercorporality are referenced in this context. See e.g. Meyer-Drawe 2008, Orlikowski 2017. To my knowledge, there are no studies in the field of educational theory which focus on Merleau-Ponty’s philosophy of science. Generally Merleau-Ponty’s thoughts on sedimentation have a great influence in discussions on embodied experience. See e.g. Wehrle and Breyer, 2016, Romdenh-Romluc 2015, Ostrow 1990.

45 However, for a productive exchange it is necessary not to confuse the way empirical sciences deal with phenomena and what they actually say about phenomena as a result of their experiments. For the later Merleau-Ponty, it is not the scientific findings, but the *praxis* in which scientists engage that is of interest for philosophy.

46 This kind of reflecting upon the body certainly does not mean to step back and think about the body in a detached way. It means to consider and ponder the involvement of the body in the process of observing practically, here and now, i.e. in a situated way. When Merleau-Ponty suggests that the reflection of the scientist is different to the one of the philosopher, this can only be explained by allowing for an understanding of reflection that is not reduced to a detached use of proposition and thought.

47 While in *Eye and Mind* (Merleau-Ponty 1993) science is taken to be nothing more than an intellectual trend, which blocks an investigation of Being by trying to objectify it, in *The Visible and the Invisible* (Merleau-Ponty 1968b) science is described as an ambivalent business departing from what Merleau-Ponty designates as perceptual faith, but trying to overcome it.

48 Merleau-Ponty spends a lot of time on thinking about a philosophical approach eligible for dealing with ontological questions. Due to the fragmentary status of *The Visible and the Invisible* (1968b) it is difficult to tell how this approach would look in detail, but it seems to be clear that Merleau-Ponty develops the conviction that it is not possible to speak about Being in a direct way. Therefore, being influenced from structuralist theories he sketches an ‘indirect’ or ‘lateral’, sometimes also ‘negative’ method. See Merleau-Ponty 1968b: 179. For further reading upon Merleau-Ponty’s late ontology see Barbaras 2004and Dastur 2001.

49 For Merleau-Ponty “primordial experience” (Merleau-Ponty 1993) is a kind of pre-ordered experience that cannot be translated into language. Aesthetic experience is exemplary for Merleau-Ponty’s understanding of primordial experience.

50 Organized and structured experience emerges thanks to sedimentation. In this respect, Dreyfus’ expert experiences in an organized and structured fashion. Merleau-Ponty’s juxtaposition of unfamiliar and familiar experience, stressing the epistemic and even ethical importance of considering unfamiliarity, hints at the limits of the Dreyfusian understanding of skillful coping.

51 I cannot elaborate on the consequences that bringing the body into intellectual engagement has for schools or other pedagogical institutions in this paper extensively. My example is intended to give an idea of one possible instance of regarding both body and mind as active players in a problem-centered learning setting. Body and mind here cooperate in an intellectual engagement, reflecting each other’s limitations and critically relating to them with the aim of finding a promising foothold for further engagement. In this respect the cooperation of body and mind can be understood as a motor for further learning on a more general level.

52 As I have already indicated in my analysis of the Austrian school curriculum, the body and the senses are not considered explicitly as valuable for teaching philosophy. Therefore current educational policy and the established ways of teaching philosophy including their grounding ideologies have to be criticized. However, I believe that it is possible to engage in alternative ways of doing philosophy in schools without rejecting the school system with its regulations entirely. Rather, it is possible to make use of the freedom given by a framework curriculum. In order to do so I regard it as necessary that people working in the educational field – theorists as well as practitioners – are aware of the possibilities as well as the limits of the policies that is supposed to shape and guide their work.

53 https://www.theguardian.com/stage/2015/apr/07/chris-thorpe-theatre-confirmation-a-nations-theatre (accessed: 4.12.2019)

54 Bringing the body into the philosophy classroom in such a way should not be restricted to a unique event. And it should not be taken to be the only way to integrate the body as an active player in intellectual engagement. Rather, for a long-term effect changes on a structural level are needed and grounding ideologies have to be rethought not only by those who work in the pedagogical field, but also by those who conceptualize educational policies.

55 Taking fiction as a trigger for learning processes clearly hints at the importance of a more encompassing understanding of aesthetic education.

56 I cannot elaborate on these challenges and how to face them at length here. Although there are many challenges bound to the postulate of bringing the body into all classrooms – and not only the gym hall or the art education studio –, I think it is possible to work towards its realization step by step.
